# Editorial: Reviews in induced pluripotent stem cells

**DOI:** 10.3389/fcell.2023.1197891

**Published:** 2023-05-04

**Authors:** Mirella Dottori, Wan-Ju Li, Gabriella Minchiotti, Alessandro Rosa, Federica Sangiuolo

**Affiliations:** ^1^ School of Medical, Indigenous and Health Sciences, University of Wollongong, Wollongong, Australia; ^2^ Department of Orthopedics and Rehabilitation, University of Wisconsin-Madison, Madison, United States; ^3^ Institute of Genetics and Biophysics “A. Buzzati-Traverso”, Consiglio Nazionale delle Ricerche, Naples, Italy; ^4^ Department of Biology and Biotechnologies “Charles Darwin”, Sapienza University of Rome, Rome, Italy; ^5^ Center for Life Nano- & Neuro-Science, Fondazione Istituto Italiano di Tecnologia (IIT), Rome, Italy; ^6^ Department of Biomedicine and Prevention, University of Rome Tor Vergata, Rome, Italy

**Keywords:** induced pluriopotent stem cells, disease model cells, organoid, precision medicine, cell differentiation

## Introduction

Induced pluripotent stem cells (iPSCs) can be generated from human adult cells, such as skin cells, by a reprogramming process that induces pluripotency. This means that iPSCs have the ability to differentiate into any type of cell in the body, just like embryonic stem cells (ESCs).

iPSCs were first generated in 2006 by Shinya Yamanaka and his team, who discovered that by introducing specific genes into adult cells, they could reprogram them into a pluripotent state (Takahashi and Yamanaka, 2006). This breakthrough discovery revolutionized the field of stem cell research, as it provided an ethical and practical alternative to using ESCs.

iPSCs have numerous potential applications in regenerative medicine, drug development, and disease modeling. They can be used to generate different cell types, such as heart cells, neurons, and liver cells, which can then be used for transplantation or for studying diseases and testing drug candidates. Moreover, iPSCs allow for the development of precision medicine approaches that aim to provide individualized medical care and treatments based on a person’s unique genetic characteristics.

Despite their tremendous potential, iPSCs still face several challenges, such as the risk of genetic abnormalities and the difficulty of controlling their differentiation into specific cell types. However, ongoing research in the field is continuously improving our understanding of iPSCs and unlocking their potential for future medical applications.

### Improving iPSC *in vitro* differentiation and maturation for disease modeling

Directing iPSC differentiation in a controlled manner remains a “Holy Grail” of stem cell research. While different strategies have been developed to achieve this goal, approaches focusing on the interaction of metabolic regulation and control of stem cell specification have attracted significant interest (Shapira and Christofk, 2020). During differentiation, iPSCs acquire more mature mitochondria and undergo a metabolic shift from glycolysis to oxidative phosphorylation (Yang et al., 2014; Teslaa and Teitell, 2015), suggesting metabolic regulation is critical to directing stem cell fates and achieving maturation of iPSC-differentiated cells (Feyen et al., 2020). For cardiomyocyte (CM) differentiation of iPSCs, metabolic switching is necessary to derive mature functional CMs (Yuan and Braun, 2017). One of the major obstacles encountered with the induction of human iPSC-derived CMs (hiPSC-CMs) *in vitro* is that using existing protocols, metabolically, structurally, and functionally immature fetal CM-like cells, instead of mature adult CMs, are generated from hiPSCs (Yang et al., 2014). To mature hiPSC-CMs, Ye et al. carried out a study to improve mitochondrial energy metabolism and maturation of differentiated CMs by activating adenosine 5′-monophosphate (AMP)-activated protein kinase (AMPK), a master regulator of cellular metabolism. They demonstrate that hiPSC-CMs treated with the AMPK activator AICAR showed increased oxidative phosphorylation and oxygen consumption rate and underwent morphological and structural maturation with the expression of CM-specific markers. Their findings provide insight into improving methods to produce mature hiPSC-CMs in culture.

The crosstalk between endothelial cells (ECs) and CMs is required for normal cardiac development, but also it is involved in the onset and progression of cardiac disease. Indeed, ECs and cardiomyocytes are in close proximity and communicate through the secretion of paracrine signals as well as direct cell-to-cell contact (Colliva et al., 2020). Helle et al. aim at elucidating the basis of organotypic development of cardiac ECs and their crosstalk with CMs, by combining co-culture of CMs and ECs derived from hiPSCs, morphological analysis and single cell transcriptomic data. The rationale of the study is that co-culture would remodel hiPSC-ECs and hiPSC-CMs transcriptomes and results in an *in vitro* model that is more relevant for cardiac modeling. In line with this hypothesis, hiPS-ECs rapidly acquire a cardiac endothelial phenotype, and increase the expression of genes related to cardiovascular development and myofibrillar contraction. These findings resemble organotypic adaptation to the requirements of the parenchymal cells, and are in line with that recently described in murine heart-derived ECs. Of relevance, while ECs transcriptome is largely remodeled by the presence of hiPSC-CMs, these cells are much less affected, suggesting high plasticity of ECs in response to paracrine signals. These findings provide a novel approach to studying the development of cardiac ECs and their interactions with CMs and offer a new *in vitro* model to study the effects of drugs and other treatments on the development of cardiac ECs.

### Towards the use of iPSCs in precision medicine

Within the last decade, the development of organoids as an *in vitro* model system has rapidly advanced across most fields in medical biology. The generation and application of organoids in research is at the forefront when considering some of the most innovative technologies developed to date in pluripotent stem cell research. In this special edition, Novelli et al. provide an excellent and comprehensive review of organoid biology, including the history of how organoids were derived and their diverse types, their classical characteristics, and their wide range of applications in research. The review highlights major studies describing how organoids have been applied to study and develop treatments for genetically inherited diseases, infectious diseases, and cancer, as well as their use in toxicology testing. The authors also cover some practical considerations of using organoids in research, such as ethical issues, and the development of ‘organoid factories’ in research centers and industry.

It is widely known that hiPSCs constitute the gold standard as a study model in the field of regenerative medicine, precision disease modeling and drug design. A very important aspect to consider in undertaking these studies consists of both the genetic background and the race of the individuals from whom these cells were derived, in order to ensure a fair representation of diseases across racial, sex and ethnic groups. As reported by Nguyen et al., to date the major hiPSC repositories in North America and Europe do not comply with these requirements. Authors have analyzed the states of diversity in hiPSC repositories and the transcriptomics profiles in hiPSC and hiPSC-derived cardiomyocytes in different races and sexes. More than half of the total cell samples banked belong to the White cohort with respect to Black one (4.5%) and the same situation is present for disease/organ types. Thus, authors emphasize the need to obtain inclusive repositories for ameliorating experimental design in disease modeling and pharmaceutical developments.

Copy number variations (CNVs), which involve differences in the number of copies of a DNA sequence, have been associated with various human diseases. For instance, CNVs affecting the CHRNA7 gene in the 15q13.3 region have been linked to several neuropsychiatric disorders. Unfortunately, animal models are limited in replicating human phenotypes due to species-specific differences in this chromosome region. Giovenale et al. provide an overview of recent publications reporting the generation and characterization of iPSCs from patients with 15q13.3 CNVs, offering a promising avenue for disease modeling and drug screening. The authors emphasize the need for common guidelines to address sources of variability among different studies, including differences in boundaries of truncations and duplications.
